# New evidence showing that the destruction of gut bacteria by antibiotic treatment could increase the honey bee’s vulnerability to *Nosema* infection

**DOI:** 10.1371/journal.pone.0187505

**Published:** 2017-11-10

**Authors:** Jiang Hong Li, Jay D. Evans, Wen Feng Li, Ya Zhou Zhao, Gloria DeGrandi-Hoffman, Shao Kang Huang, Zhi Guo Li, Michele Hamilton, Yan Ping Chen

**Affiliations:** 1 USDA-ARS Bee research Laboratory, Beltsville, MD, United States of America; 2 College of Bee Science, Fujian Agriculture and Forestry University, Fuzhou, China; 3 Institute of Apicultural Research, Chinese Academy of Agriculture Sciences, Beijing, China; 4 USDA-ARS Carl Hayden Bee Research Center, Tucson, AZ, United States of America; University of North Carolina at Greensboro, UNITED STATES

## Abstract

It has become increasingly clear that gut bacteria play vital roles in the development, nutrition, immunity, and overall fitness of their eukaryotic hosts. We conducted the present study to investigate the effects of gut microbiota disruption on the honey bee’s immune responses to infection by the microsporidian parasite *Nosema ceranae*. Newly emerged adult workers were collected and divided into four groups: Group I—no treatment; Group II—inoculated with *N*. *ceranae*, Group III—antibiotic treatment, and Group IV—antibiotic treatment after inoculation with *N*. *ceranae*. Our study showed that *Nosema* infection did not cause obvious disruption of the gut bacterial community as there was no significant difference in the density and composition of gut bacteria between Group I and Group II. However, the elimination of gut bacteria by antibiotic (Groups III and IV) negatively impacted the functioning of the honey bees’ immune system as evidenced by the expression of genes encoding antimicrobial peptides *abaecin*, *defensin1*, and *hymenoptaecin* that showed the following ranking: Group I > Group II > Group III > Group IV. In addition, significantly higher *Nosema* levels were observed in Group IV than in Group II, suggesting that eliminating gut bacteria weakened immune function and made honey bees more susceptible to *Nosema* infection. Based on Group IV having displayed the highest mortality rate among the four experimental groups indicates that antibiotic treatment in combination with stress, associated with *Nosema* infection, significantly and negatively impacts honey bee survival. The present study adds new evidence that antibiotic treatment not only leads to the complex problem of antibiotic resistance but can impact honey bee disease resistance. Further studies aimed at specific components of the gut bacterial community will provide new insights into the roles of specific bacteria and possibly new approaches to improving bee health.

## Introduction

Gut commensal bacteria, also known as microbiota, are microorganisms that colonize the digestive tracts of different animal species including insects. Some of the microbiota are opportunist species whose quantity and composition vary with food sources, season and other environmental factors [1–4]. Others are stable and widespread colonists that regulate many aspects of host physiology and fitness including nutrition, detoxification, development, and resistance to pathogenic infections [5–7]. These bacteria are integrated into the biological system of their hosts and as such can be considered as a bacterial organ.

Advances in high-throughput sequencing technology have allowed a more comprehensive investigation of the community and function of gut bacteria in European honey bees *Apis mellifera* [8–14]. In honey bees, newly emerged workers contain few or no core gut bacteria. Workers obtain their gut microbiota at 3–5 days after eclosion [4, 11, 13, 15]. Gut bacteria is transmitted and shared by the colony members through the fecal-oral route, oral trophallactic interaction, consumption of bee bread, encounters with old bees within the hive, and contact with hive materials during the early adult stage [11, 13, 15]. Honeybee gut microbiota is mainly in the ileum/rectum of hindgut and most bacteria found in the midgut are located in the region of the pylorus which separates the midgut from the hindgut [13]. Compared with the gut microbiota of other animals, honey bees harbor a relatively simple, but specialized gut microbiota community that is dominated by approximately nine bacterial species or phylotypes that have not been found in environments outside of the bee gut [15]. Of the major bacterial phyla (*Firmicutes*, *Proteobacteria*, and *Actinomycetes*) found in the gut of adult worker bees, three Gram-negative species, *Gilliamella apicola*, *Frischella perrara*, and *Snodgrassella alvi* (within the phylum *Proteobacteria*) dominate in the hindgut ileum, and three Gram-positive species, *Bifidobacterium asteroides* (within the phylum *Actinomycetes*), Firm 4 and Firm5 (within the phylum *Firmicutes*) dominate in the hindgut rectum [15]. The species from the phylum *Proteobacteria* (Alpha-2.1, Alpha-2.2, and Alpha-1), and the phylum *Firmicutes* (*Lactobacillus kunkeei*) dominate in the crop of the foregut are also present in the hive and colony food including nectar, honey and beebread [15]. Bacterial communities in the foregut and midgut are significantly lower in number and diversity than that of the hindgut [11, 13]. Previous studies confirmed that these bacterial species are largely restricted to honeybees and other bee species including the Asian honey bees *A*. *cerana* and some bumble bee species[10], reflecting a long-term coevolutionary history of the sister bee species [15, 16].

Insects including honey bees lack acquired immune response but have evolved effective immune responses to combat infection by pathogens [17–19]. A previous study on *Drosophila melanogaster* reported that gut bacteria could regulate host innate immunity, and affect susceptibility to pathogens or parasites [20]. A similar phenomenon was reported in bumble bees, close cousins of honey bees [21–23]. Recent research has led to important insights into the roles of the gut microbiota in physiology, immunity, behavior, growth, development, and survivor of the honey bee host [24, 25]. A study by Kwong et al. [24] reported that the expression of AMPs including *apidaecin* and *hymenoptaecin* in gut tissue of honey bees was upregulated when the microbiota was present and higher apidaecin concentrations were found in bees harboring the normal gut microbiota than in bees lacking gut microbiota. Raymann et al. (2017) reported that gut bacteria perturbation by antibiotics could increase the susceptibility to opportunistic bacterial pathogens and decreased the survivorship of honey bees [26], providing additional evidence that the gut microbiome plays an important role in honey bee immune regulation. Further studies showed that gut microbiota could affect honey bee growth, hormonal signaling, behavior, sugar metabolism, and pollen digestion [8, 25, 27], revealing diverse metabolic functions of gut bacteria that are likely to contribute to bee growth and health.

*Nosema*. *ceranae* is an obligate intracellular fungal pathogen of the honey bee and causing a serious disease known as nosemosis that is characterized by digestive tract dysfunction and consequent metabolic disorders and sometimes immune suppression [28, 29]. Since its emergence as a virulent pathogen of *A*. *mellifera*, *N*. *ceranae* has often been invoked in honeybee colony losses worldwide [30–33]. In this study, we conducted an investigation to explore the effects of gut microbial communities on honeybee susceptibility to *Nosema* infection by disturbing the microbiome with antibiotic, and then infecting the bees with *N*. *ceranae*. We found that the disruption of gut bacteria by antibiotic could cause the inhibition of honey bee immunity and make honey bees more susceptible to *Nosema* infection, thereby shortening their lifespan.

## Material and methods

### Ethics statement

No specific permits were required for the described studies. Observations were made in the USDA-ARS Bee Research Laboratory apiaries, Beltsville, Maryland, USA; therefore, no specific permissions were required for these locations. The apiaries are the property of the USDA-ARS and are not privately-owned or protected. Studies involved the European honey bee (*Apis mellifera*), which is neither an endangered nor protected species.

The experimental honey bee colonies established from commercial packages were reared in the Bee Research Laboratory apiaries. These colonies were naturally overwintered, and allowed for the natural replacement of queens that were open-mated when necessary.

### *Nosema ceranae* spores

*N*. *ceranae* spores were purified for bee inoculation according to methods described previously [34, 35]. Infected adult workers were sampled from colonies identified as heavily infected by *N*. *ceranae* based on a monthly survey for spore collection. The midgut of an individually infected honey bee was removed by pulling on the last abdominal segment of the bee with forceps until the midgut was separated from the abdomen. Midguts were homogenized in sterile distilled water using a glass homogenizer. After filtering through a nylon mesh (65 μm pore size) to remove larger fragments, the homogenate was centrifuged for 5 minutes at 3,000 g and the supernatant was discarded. The spore-containing pellet was completely resuspended in sterile water and centrifuged for 10 min at 5,000 g. This process was repeated twice. The spore-containing pellet was suspended in distilled water and stored at room temperature to be used within one week. The concentration of the spore suspension was determined using a hemocytometer under light microscopy as previously described [36]. The spore suspension was diluted to a concentration of 5×10^7^ using a 50% sugar solution (W /V) for inoculation.

### Honey bee samples and experimental setup

Bee colonies used in the experiment were screened monthly for pathogens and parasites by molecular methods and light microscopy. Frames with emerging brood and food stores were removed from colonies identified as free of *Nosema* infection by monthly surveys. Briefly, 30 abdomens of bees collected at the entrance of the colony were dissected and ground up thoroughly in 30 ml of deionized H_2_O. 10 μl of the homogenate was loaded onto a hemocytometer and the presence of spores was determined under light microscopy following a previously described method [36]. Brood frames from colonies with no apparent *N*. *ceranae* infection were individually placed in a mesh-walled cage and incubated in an insect growth chamber at 34 ^o^C and 55% relative humidity. Newly emerged bees roamed the frame for 48 hours so they could be naturally inoculated by residual gut symbionts on the frame surface. The individual bees were collected and transferred to laboratory bee rearing cages (40 bees per cage) and kept in an insect growth chamber as described [37].

Rearing cages were divided into four experimental groups (six cages/group) and subjected to the following treatments: Group I, bees were fed with 50% sugar solution (W/V) (negative control); Group II, bees were inoculated with *N*. *ceranae* spores by individually feeding 2 μl spore suspensions in 50% sugar solution using a pipette with a 10 μl filtered pipette tip (1×10^5^ spores/per bee); Group III, bees were fed with 50× dilution of penicillin-streptomycin (Thermo Fisher scientific, Inc.) in 50% sugar solution (W /V) for the experimental period; and Group IV, bees were inoculated with *N*. *ceranae* spores following the same methods described in Group II and then treated with antibiotic following the same methods described for Group III. Each rearing cage was equipped with one feeder which was filled with 50% sugar solution and supplied with patties of bee-collected pollen to provision the bees *ad libitum*.

For each treatment group, three cages were used and the number of dead bees were counted daily for determining the life span of the different treatment groups. Observations lasted until all bees were dead. Three cages were used for sampling honey bees at Day 3, Day 7 and Day 11 post treatment. Eight live bees were collected at each time point and immediately placed into a -80 ^o^C freezer for subsequent DNA and RNA extraction.

### DNA and RNA extraction

Four bees collected at each time point from each cage were used for individual DNA isolation, (N = 4 bees x 3 cages). DNAzol (Invitrogen, Carlsbad, CA) was used for DNA isolation from the collected honey bees according to the manufacturer's instructions. Four bees collected at each time point from each cage were used for individual RNA isolation (N = 4 bees X 3 cages). TRIzol (Invitrogen, Carlsbad, CA) was used for RNA extraction following the manufacturer's instructions. The resultant RNA pellets were resuspended in UltraPure DNase/RNase-Free water with the addition of Ribonuclease Inhibitor (Invitrogen, Carlsbad, CA). The quantity and purity of both DNA and RNA were measured using a NanoDrop 8000 Spectrophotometer (NanoDrop Technologies, Wilmington, DE). DNA samples were stored at -20°C and RNA samples were stored at -80°C until used.

### qPCR

To determine the diversity and composition of gut bacteria in honey bees not exposed to antibiotics, the relative abundance of gut bacterial taxa in Groups I and II were analyzed by qPCR using bacteria 16s rDNA universal primers and phylotype-specific 16s rDNA primers [38, 39] (Table 1). Levels of *Nosema* in Groups II and IV were measured by qPCR method using *N*. *ceranae* rDNA primers to determine the effects of antibiotic treatment on honeybees’ susceptibility to *Nosema* infection [30].

**Table 1:** Primer used for quantitation the total and phylotype specific bacterial and *N*. *ceranae* by qPCR.

Target	Sequence (5' to 3')	Reference
universal bacteria	F: AGAGTTTGATCCTGGCTCAG R: CTGCTGCCTCCCGTAGGAGT	Schwarz *et al*., 2016 [38]
*Snodgrassella alvi*	F: CTTAGAGATAGGAGAGTG R: TAATGATGGCAACTAATGACAA	Schwarz *et al*., 2016 [38]
*Gilliamella apicola*	F: GTATCTAATAGGTGCATCAATT R: TCCTCTACAATACTCTAGTT	Schwarz *et al*., 2016 [38]
*а-Proteobacteria*	F: CIAGTGTAGAGGTGAAATTC R: CCCCGTCAATTCCTTTGAGTT	Bacchetti *et al*., 2011 [39]
*г-Proteobacteria*	F: TCGTCAGCTCGTGTYGTGA R: CGTAAGGGCCATGATG	Bacchetti *et al*., 2011 [39]
*Bacteroidetes*	F: CRAACAGGATTAGATACCCT R: GGTAAGGTTCCTCGCGTAT	Bacchetti *et al*., 2011 [39]
*Firmicutes*	F: TGAAACTYAAAGGAATTGACG R: ACCATGCACCACCTGTC	Bacchetti *et al*., 2011 [39]
*Actinobacteria*	F: TACGGCCGCAAGGCTA R: TCRTCCCCACCTTCCTCCG	Bacchetti *et al*., 2011 [39]
*Nosema cerana*	F: CGGATAAAAGAGTCCGTTACC R: TGAGCAGGGTTCTAGGGAT	Chen *et al*., 2008 [30]

qPCR was run on a CFX384 Touch Real-Time PCR System (Bio-Rad, Hercules, CA). The Brilliant III Ultra-Fast SYBR Green QPCR Mix (Agilent, Santa Clara, CA) was used for setting up the qPCR reactions consisting of 5 μl 2×qPCR mix, 0.25 μl of each forward and reverse primers (20 uM), 0.5 μl DNA, and 4 μl nuclease-free water. PCR amplification consisted of the following steps: 95°C for 3 min, followed by 40 cycles of 95°C for 20 s, 55°C for 25 s, and 72°C for 30 s and incubation at 72°C for 10 min. After amplification, a dissociation curve was constructed using 81 complete cycles of incubation where the temperature was increased by 0.5°C/cycle, beginning at 55°C and ending at 95°C to verify specificity of the primers.

### qRT-PCR

To determine the effect of antibiotic treatment on gut bacteria activity, expression levels of bacteria 16S rDNA in bees from Group I and Group III were measured by qRT-PCR with bacteria 16S rDNA universal primers. The qRT-PCR method was also used to measure the expression of genes encoding antimicrobial peptides *abaecin*, *defensin1*, and *hymenoptaecin* in bees from all experimental groups (Group I, II, III, and IV). The expression of a housekeeping gene, *β*-actin, in each sample was also measured for normalization of the qRT-PCR results. The primers used for measuring expression of the bacteria 16S rDNA, immune genes, and housekeeping gene were previously reported [17, 38, 40] (Table 2). Total volume of the qRT-PCR reaction was 12.5 μl consisting of 6.25 μl of 2×Brilliant II SYBR Green qRT-PCR 1-Step Master Mix (Agilent, Santa Clara, CA), 0.375 μl each of forward and reverse primers, 0.5 μl of RT/RNase block enzyme mixture, and 0.5 μl of total RNA. qRT-PCR was carried out in a CFX384 Touch Real-Time PCR System (Bio-Rad, Hercules, CA) with the following thermal profile: 50°C for 30 min, 95°C for 10 min, followed by 40 cycles of 95°C for 30 s, 59°C for 60 s, and 72°C for 60 s, then 72°C extension for 10 min. A dissociation curve was added at the end of PCR cycling as described for the qPCR method above.

**Table 2:** Primer used for measuring expression level of genes encoding *β-actin*, universal 16S rDNA bacteria, and antimicrobial peptides (*abaecin*, *defensin1* and *hymenoptaecin*) by qRT-PCR method.

Gene name	Sequence (5' to 3')	Product size (bp)	Reference
*β-actin*	F: TTGTATGCCAACACTGTCCTTT R: TGGCGCGATGATCTTAATTT	120	Simone et al., 2009 [40]
univeral bacteria	F: AGAGTTTGATCCTGGCTCAG R: CTGCTGCCTCCCGTAGGAGT	328	Schwarz et al., 2016 [38]
*abaecin*	F: CAGCATTCGCATACGTACCA R: GACCAGGAAACGTTGGAAAC	72	Evans, 2006 [17]
*defensin1*	F: TGCGCTGCTAACTGTCTCAG R: AATGGCACTTAACCGAAACG	119	Evans, 2006 [17]
*hymenoptaecin*	F: CTCTTCTGTGCCGTTGCATA R: GCGTCTCCTGTCATTCCATT	200	Evans, 2006 [17]

### Data analysis

The comparative Ct method (ΔΔ Ct Method) [41] was used to compare the level of gut bacteria activity, *Nosema* and expression of genes encoding antimicrobial peptides at each time point post treatment. For each target gene at each time point post treatment, the quantity or level was quantified based on the value of the cycle threshold (Ct) which represents the number of cycles needed to generate a fluorescent signal above a predefined threshold and was expressed as means + SE. The mean value and standard deviations of the target gene were normalized using the Ct value corresponding to the measurements of endogenous control, *β-actin* to yield ΔCt. At each time point post treatment, the group that had the minimal expression level of the target gene was chosen as a calibrator. The ΔCt value of each group was subtracted by ΔCt value of the calibrator to yield ΔΔCt. The concentration of the target among different experimental groups relative to the calibrator at each time point was calculated using the formula 2^-ΔΔCt^ and expressed as the fold-difference.

The comparative Ct method also was used to interpret the relative abundance of bacteria taxa (*Snodgrassella alvi*, *Gilliamella apicola*, *а-Proteobacteria*, *г-Proteobacteria*, *Bacteroidetes*, *Firmicutes*, *Actinobacteria*) in bees from Group I and Group II (no antibiotic treatment). Bees from Groups III and IV (with antibiotic treatment) had Ct values from PCR amplification using 16S rDNA universal primers and phylotype-specific 16s rDNA primers that were above 30 and considered as undetectable Ct values. Therefore, the groups of bees treated with antibiotic were given a value of 1 (one) and used as a calibrator for each primer pair. The relative abundance of total bacteria and specific bacterial phylotypes at each time point post treatment was expressed as an n-fold difference relative to the calibrator.

The statistical significance of relative quantification of each target gene at each time point post treatment among the different groups was analyzed by using SPSS (PASW Statistics 18, SPSS Inc.). The independent t-test was used to determine whether the mean difference in concentration of bacterial 16S rRNA or *Nosema* rDNA between two experimental groups was statistically significant (p ≤ 0.05). One–Way ANOVA was used to test for differences in the expression level of antimicrobial peptides *abaecin*, *defensin1*, and *hymenoptaecin* among the four experimental groups.

Survivorship among the different treatments was analyzed using the Kaplan-Meier method. Pairwise comparisons were carried out using Log-rank test. In all cases, *P* ≤ 0.05 was considered to be significant.

## Results

### Antibiotic caused significant disruption of bacterial activity in honey bees

**Fig 1:**
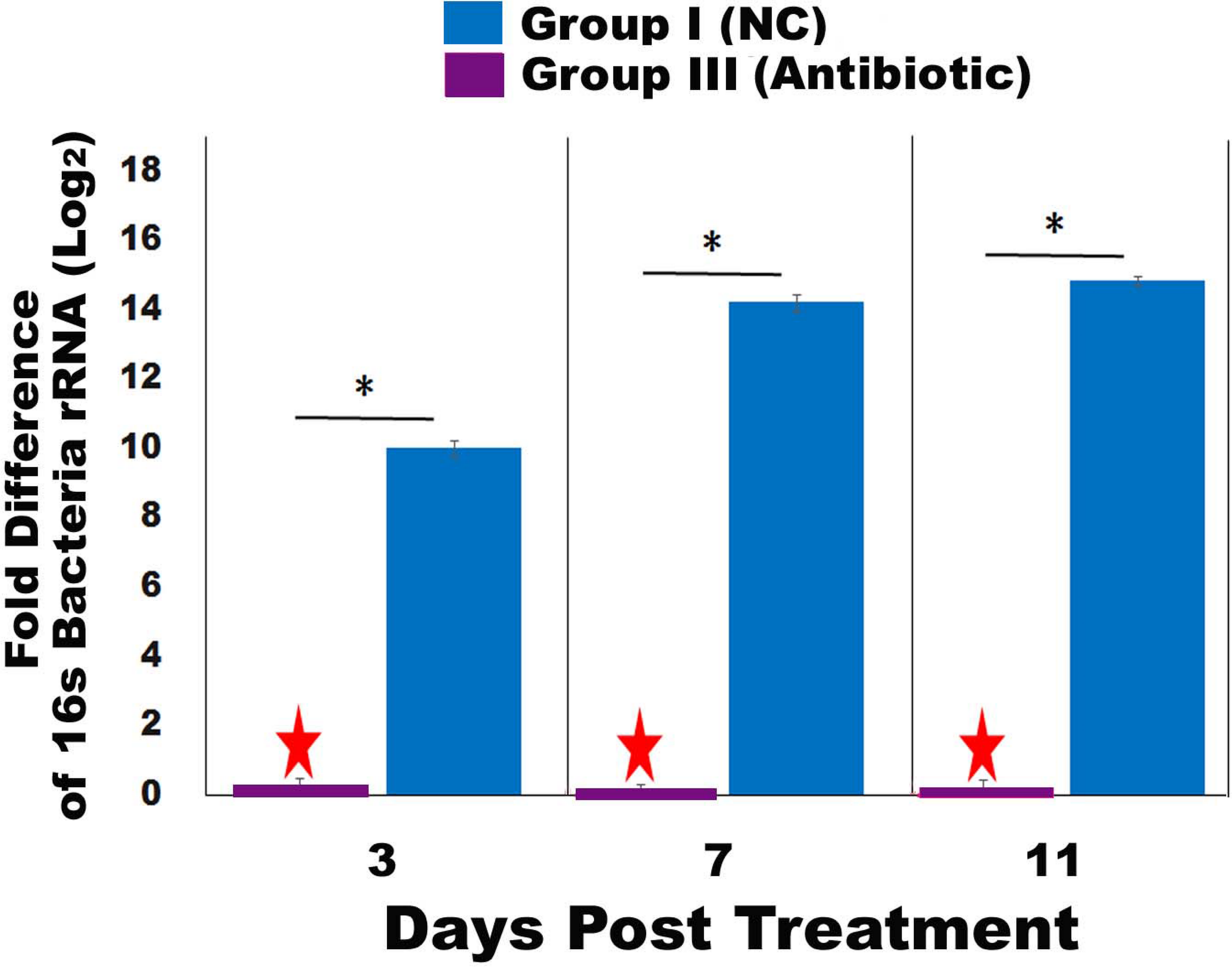
Disruption of bacterial activity in honey bee by antibiotic treatment. Bacteria activity of honey bees from the negative control group without any treatment (Group-I) and the group treated with antibiotic (Group-III) at Day 3, Day 7, and Day 11 post treatment. The relative gene expression of 16S rRNA transcript of bacteria in honey bee in Group-III was significantly suppressed compared to Group-I, thereby serving as a calibrator at each time point post treatment (marked by a star). The X axis indicates the days post treatment. The Y axis depicts the Log_2_ fold difference of 16S rRNA expression in Group-I relative to the calibrator. The relative fold difference of gene expression levels of 16S rRNA are expressed as mean ± SE. Significant differences by independent samples *t*-test between Group-I and Group-III are indicated by asterisks (*P* ≤0.05).

Antibiotic treatment significantly disrupted the bacterial activity in honey bees. The average expression level of bacteria 16S rRNA in Group I was significantly higher than that in Group III at day 3, day 7, and day 11 post treatment, respectively (Day-3, *t* = -11.946, df = 11.432, *P* < 0.001; Day-7, *t* = -26.854, df = 22, *P* < 0.001; Day 11, *t* = -17.654, df = 11.526, *P* < 0.001) (Fig 1). Also, the fold change in terms of 16s rRNA expression between Group I and Group II was significantly greater at day 7 and day 11 than at day 3 (Fig 1) (S1 Table).

### *Nosema* infection does not significantly impact the abundance and type of bacteria in honey bee

**Fig 2:**
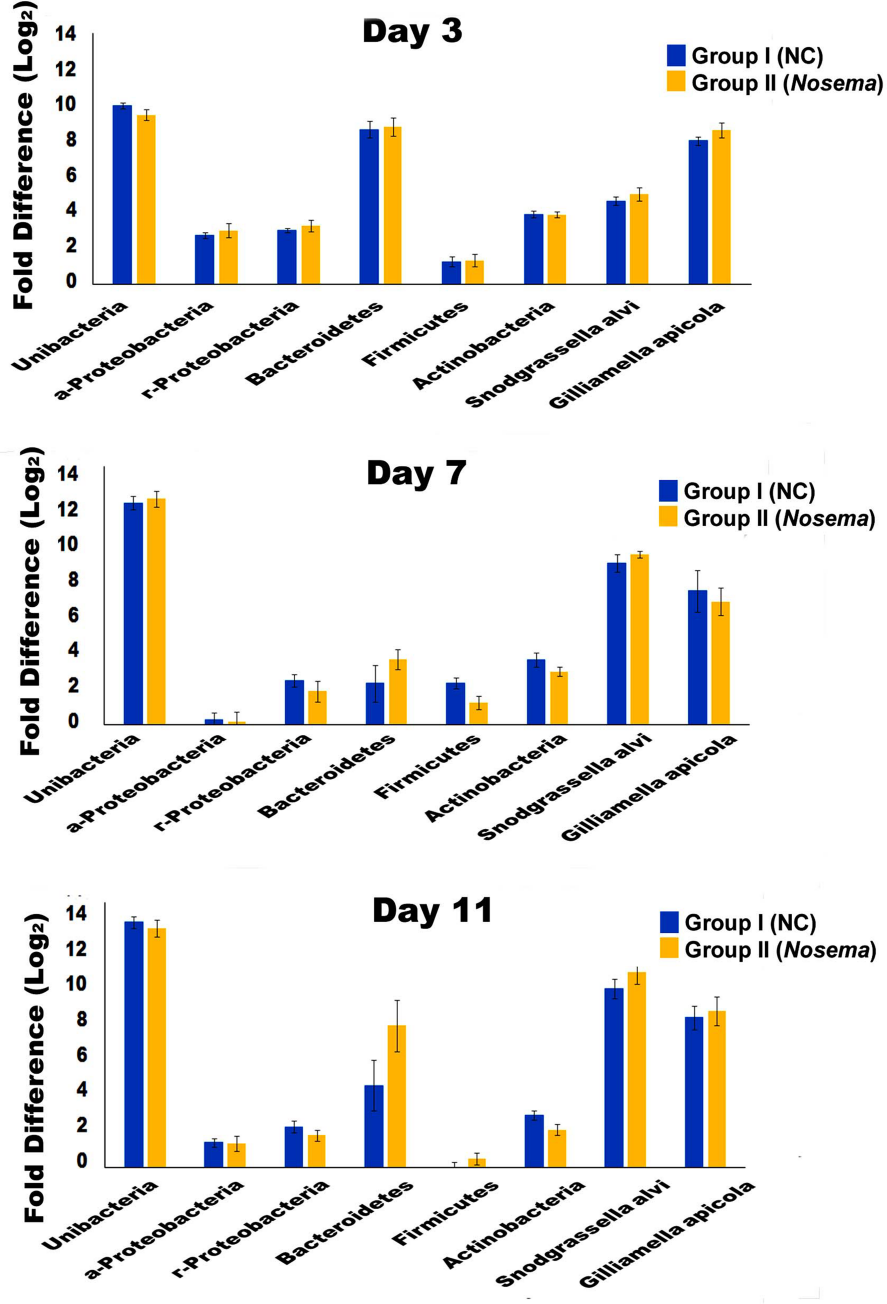
The relative abundance of the total bacteria and specific bacteria phylotypes. The relative quantifiation of the total bacteria and specific bacteria phylotypes including *а-Proteobacteria*, *r-Proteobacteria*, *Bacteroidetes*, *Firmicutes*, *Actinobacteria*, *Snodgrassella alvi*, *Gilliamella apicola* in Groups I and Group II without antibiotic treatment was estimated by qPCR with 16S rDNA universal primers and phylotypes specific 16S rDNA primers individually. The bees from Group III and IV with antibiotic treatment had an undetectable Ct values and were given a value of 1 (one) and used as a calibrator for each primer pair. The relative abundance of total bacteria and specific bacterial phylotypes at each time point post treatment was expressed as an n-fold difference relative to the calibrator. The X axis indicates the days post treatment. The Y axis depicts the Log_2_ fold difference of total bacteria and specific bacteria phylotypes in Group I and Group II relative to the calibrator. The relative fold difference of each target is expressed as mean ± SE. Significant differences by Paired *t*-test between Group-I and Group-III at each time point are indicated by asterisks (*P* ≤0.05) (Supplementary Material). There was not statistically significant difference in the quantity of total bacteria and seven phylotypes between Group I and Group II at day 3, day 7 and day 11 after eclosion, suggesting that *Nosema* infection did not significantly impact the abundance and type of bacteria in honey bees.

The relative quantification of the total bacteria and specific bacteria phylotypes in Group I and Group II without antibiotic treatment confirmed the presence of bacterial phylotypes inhabiting the honey bee but with the bacterial composition differing among time points post treatment (Fig 2). The qPCR quantification of total bacteria and specific bacteria phylotypes showed no statistically significant difference in the quantity of total bacteria between Group I and Group II at day 3, day 7 and day 11 after eclosion (Paired *t*-test, *t* = 0.948, df = 2, *P* = 0.443). Moreover, there was no statistical difference in the quantity of the seven phylotypes between Group I and Group II at day 3, day 7 and day 11 after eclosion (Paired *t*-test, Day 3: *t* = 1.286, *P* = 0.239; Day 7, *t* = 0.442, *P* = 0.672; Day 11, *t* = 0.951, *P* = 0.373), suggesting that *Nosema* infection did not significantly impact the abundance and type of bacteria in honey bees (S2 Table).

### The disruption of bacteria in honey bees by antibiotic treatment could negatively impact the expression levels of genes encoding antimicrobial peptides *abaecin*, *defensin1*, and *hymenoptaecin*

**Fig 3:**
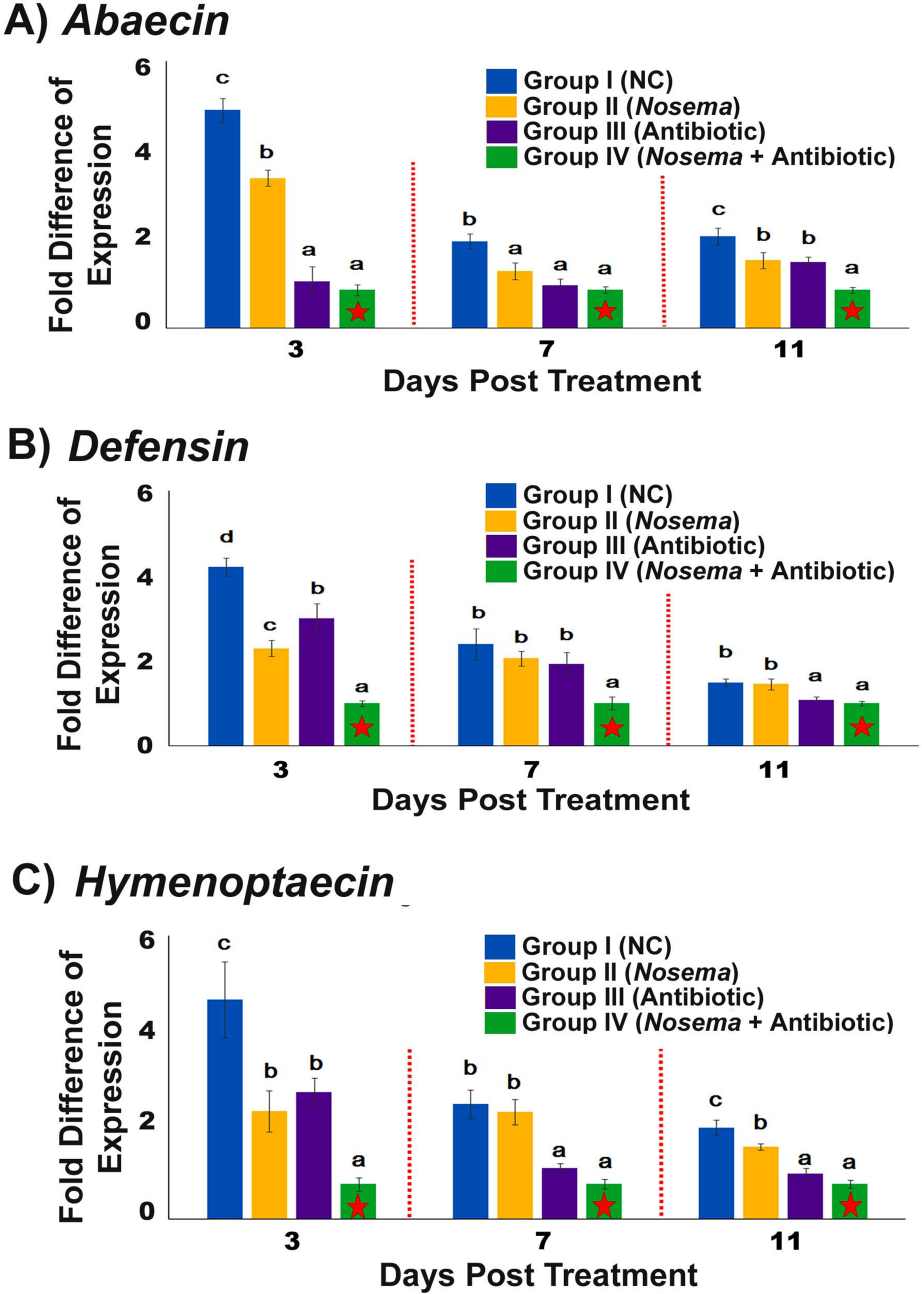
Effect of bacteria disruption on the expression of genes encoding antimicrobial peptides (AMPs). The relative expression levels of *abaecin* (A), *defensin1* (B) and *hymenoptaecin* (C) were determined by qRT-PCR from the RNA extracted from 12 honey bee per group at each time point, which showed significant differences across four experimental groups at each time point post treatment. Significant differences by One–Way ANOVA among the Group-I, Group-II, Group-III and Group-IV at each time point are indicated by asterisks (*P* ≤0.05) (Supplementary Material). The X axis indicates the days post treatment. The Y axis depicts the fold difference of expression relative to the calibrator. For each AMP, the lowest expression level was observed in Group IV at each time point post treatment among four experimental groups and therefore was chosen as a calibrator (marked by a star). The relative expression levels of other three groups at each time point was compared with calibrator and expressed as n-fold difference. The Y axis depicts fold difference relative to the calibrator. The relative fold difference of gene expression levels of each gene is expressed as mean ± SE. Different letters above each bar indicate statistically significant difference (*P* ≤ 0.05).

The fold change values of expression levels of genes encoding antimicrobial peptides (AMPs) *abaecin*, *defensin1*, and *hymenoptaecin* showed significant differences across the four experimental groups at each time point post treatment (Fig 3) (One–Way ANOVA, 3 days post treatment: for *abaecin*, F_3,47_ = 38.935, *P*<0.001; for *defensin1*, F_3,47_ = 33.748, *P*<0.001; for *hymenoptaecin*, F_3,47_ = 10.074, *P*<0.001; 7 days post treatment: for *abaecin*, F_3,47_ = 6.403, *P* = 0.003; for *defensin1*, F_3,47_ = 5.216, *P* = 0.004; for *hymenoptaecin*, F_3,47_ = 15.140, *P*<0.001; 11 days post treatment: *abaecin*, F_3,47_ = 10.078, *P*<0.001; for *defensin1*, F_3,47_ = 6.110, *P* = 0.002; for *hymenoptaecin*, F_3,47_ = 24.126, *P*<0.001) (S3 Table). For each AMP, the fold change values at each time point post treatment showed the following ranking: Group I > Group II > Group III > Group IV. Group IV had the lowest AMPs expression levels at each time point, clearly indicating that bees with antibiotic treatment and *Nosema* infection were in an immune-suppressed state. Expression level of each AMP in Group I was the highest among the four groups at each time point. Except for Day-3 post treatment, the expression levels of each AMP in Group II was higher than that in Group III, indicating that the magnitude of the negative impacts on honey bee immune responses caused by antibiotic treatment was higher than that caused by *Nosema* infection. For each experimental group, similar expression profiles were observed for each AMP. The fold change of transcript levels of *abaecin*, *defensin1*, and *hymenoptaecin* revealed a high level at Day-3 post treatment that declined thereafter and remained relatively constant over the eleven-day time-course of the experiment.

### The disruption of bacteria in the honey bee by antibiotic treatment could increase the honey bee’s susceptibility to *Nosema* infection

**Fig 4:**
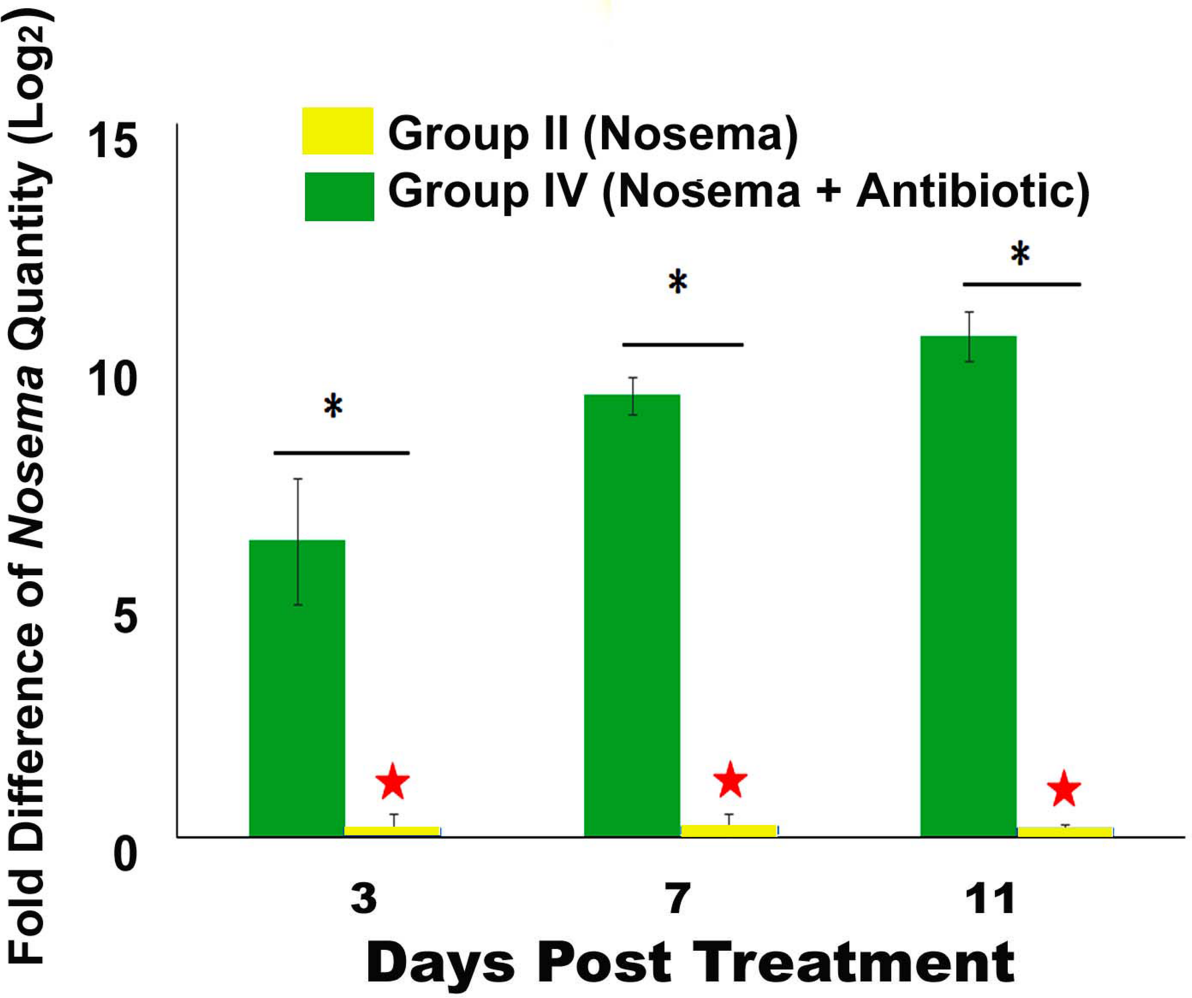
The relative quantities of *N*. *ceranae* in infected bees. Quantities of N. *ceranae* from honey bees in Group II and Group IV were determined by qPCR. The result showed that the *N*. *ceranae* quantities from honey bees in Group II was the significanly lower than that in Group IV at each time point post treatment, and therefore was chosen as a calibrator (marked by a star). The X axis indicates the days post treatment. The Y axis depicts the Log_2_ fold difference of the relative quantities of *N*. *ceranae* in Group IV relative to the calibrator. The relative fold difference of each target is expressed as mean ± SE. Significant differences by independent samples *t*-test between Group-II and Group-IV at each time point are indicated by asterisks (*P* ≤ 0.05).

The relative quantities of *N*. *ceranae* in infected bees in Group IV was signficantly higher than that in Group II at each time point post treatment (*t*-test, Day-3, *t* = 4.464, df = 10.256, *P* = 0.001; Day -7, *t* = 14.105, df = 16, *P*<0.001; Day-11, *t* = 19.407, df = 16, *P*<0.001) (S4 Table). The *Nosema* proliferated steadily over the course of time of the experiment in infected bees of Group IV in relation to the titer of *Nosema* in bees of Group II, indicating that the bacteria in honey bee could play a significant role in the honey bee’s resistance to the growth and replication of *N*. *ceranae (*Fig 4).

### Antibiotic treatment and *Nosema* infection could cause cumulative and synergistic detrimental effects on the lifespan of honey bees

**Fig 5:**
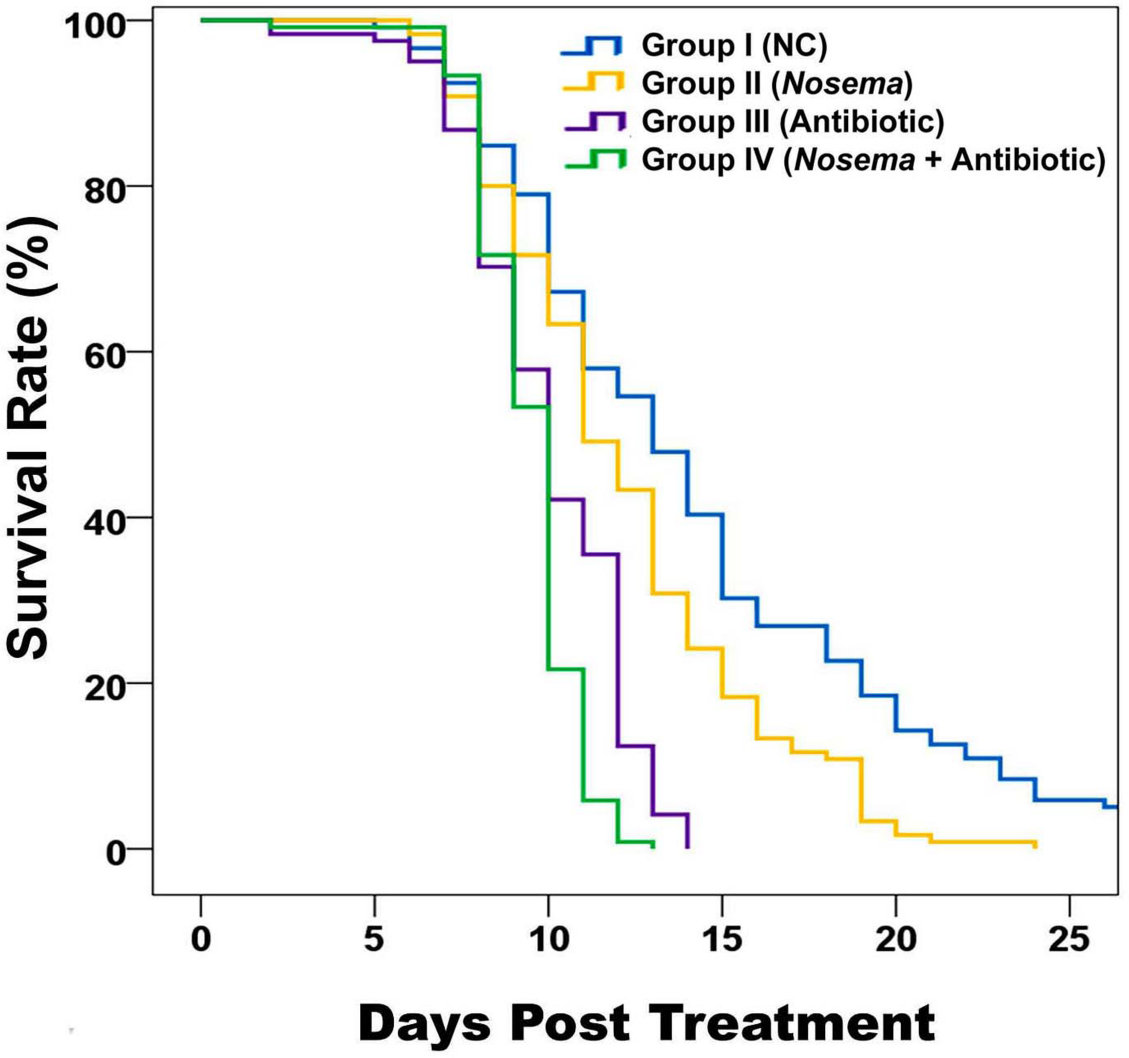
Effect of bacteria disruption by antibiotic and/or *Nosema* infection on the survivorship of adult workers. The X axis indicates the days post treatment. Y axis indicates survival rate (%) based on the daily accumulate mortality. Significant differences among the different groups were analyzed using the Kaplan-Meier method. Log-rank was used to assess the overall homogeneity between the treatments. Pairwise comparisons were carried out using Log-rank test in SPSS 18.0. In all cases, *P* ≤ 0.05 was considered to be significant. The survivor rate and longest survivor time displayed the following ranking: Group I > Group II > Group III > Group IV.

Of the four groups, the bees in Group I had the highest survival rate and longest survival time while bees in Group IV had the lowest (Fig 5). Bees in Group III and IV lived for a significantly shorter time than bees in Groups I and II (Group I vs Group III, Log-rank test: χ2 = 58.906, *P* <0.001; Group II vs Group IV, Log-rank test: χ2 = 59.528, *P* <0.001), suggesting that the antibiotic treatment alone could significantly reduce the survivorship of honey bees. *Nosema* infection influenced the survivorship of the honey bees, as seen by the statistically significant differences between Group I and Group II as well as between Group III and Group IV (Log-rank test: Group I and Group II, χ2 = 12.089, *P* = 0.001; Group III and Group IV, χ2 = 15.695, *P* <0.001).

## Discussion

Honey bees are the most important insect pollinator for agricultural crops worldwide. However, honey bees suffer from a broad range of pathogens and parasites and the population of honey bees has declined considerably in recent years [42–45]. While there is growing evidence that gut microbiota play a crucial role in regulating host metabolic and immune functions [21, 46–50], the role of gut microbiota on the honeybees’ immune responses to the microsporidia *Nosema* infection is not known in detail. As a result, we investigated the effects of gut bacteria disruption on the honey bees’ susceptibility to *Nosema* infection. Our results demonstrated that antibiotic treatment in combination with stress associated with *Nosema* infection could significantly and negatively impact the survivorship of honey bees. We hope the insight gained from this study will contribute to our understanding of the functional capacity of the gut microbiome in honey bees and improve the health of honey bees and other pollinator insects [51].

The specialized gut microbial community of honey bees has been explored and comprehensively reviewed by Kwong and Moran in 2016 [15]. Our study of gut bacterial composition and diversity confirmed the presence of the abundant bacterial phylotypes inhabiting honey bee guts [15]. The role of gut microbiota in regulating the honey bees’ susceptibility to *N*. *cerana* infection was demonstrated by the significantly lower *Nosema* loads at all three time points post treatment in honey bees with undisrupted gut bacteria compared with those treated with antibiotic. The gut microbiota-mediated host immune regulation especially by initially colonized gut bacteria was reported previously in honey bee, mice and humans [24, 25, 38, 52–56], suggesting that the acquisition and establishment of bacteria population occurs shortly after birth or eclosion. The active role of gut bacteria in maintaining health was further demonstrated by the reduced lifespan of bees treated with antibiotic, and a further decrease with *Nosema* infection. This result is consistent with a previous report and suggests that gut bacteria not only protect hosts from disease infection, but also can impact host life expectancy [26].

In general, there are two mechanisms that are utilized by gut bacteria to reduce host susceptibility to microbial infections: direct antagonistic interactions and indirect host immune regulation [57, 58]. *N*. *ceranae* is a unicellular fungal parasite that infects and accumulates in mid-gut epithelial cells of honey bees. The gut bacterial community in the hindgut of honey bee adult workers accounts for ~95% of all gut bacteria, while the crop (honey stomach or foregut) is colonized by a small population of bacteria [13, 16]. Niche separation suggests that there are minimal direct interactions between the *Nosema* and gut bacteria, which might be the reason why *Nosema* infection had no obvious effects on gut bacteria quantity and community in our study. Therefore, the increased susceptibility of honey bees to *N*. *ceranae* infection after disruption of gut bacteria was likely a result of altered honey bee immunity. While it is unclear if antibiotics exert direct effects on honey bee immune system, it is well documented that antibiotics cause rapid and profound alterations of gut microbiota, which in turn leads impaired immunity and produces long-lasting deleterious effects for the hosts.

AMPs are small molecular weight proteins with broad spectrum antimicrobial activity, thereby are a crucial part of the innate immune response found among all classes of life. Insects are one of the major sources of AMPs which are abundant in hemolymph and serve as a potent innate immune defense against diverse microbial pathogens [18, 59, 60]. *Abaecin*, *defensin1* and *hymenoptaecin* are families of AMPs that are inducible and controlled by Toll and Imd /JNK signaling pathways and have been described in honey bees [17]. By determining the expression of genes encoding *abaecin*, *defensin1* and *hymenoptaecin* after validating the successful depletion of gut bacteria by antibiotic treatment, we found gene expression levels of the three AMPs all significantly down-regulated in bees treated with antibiotic, compared to the negative control. This result is consistent with previous studies [24–26, 28, 29] and provides additional experimental evidence that gut bacteria are closely linked to the proper functioning of the immune system and the depletion of gut bacteria could lead to negative regulation of the innate immune response in honey bees.

*Nosema* is a virulent honey bee pathogen that has been suggested as one of the key contributors to increased honey bee colony losses worldwide [30–33, 61]. Antibiotics have been widely used in the field for the control of nosemosis, a honey bee disease caused by *Nosema*. The only registered treatment for *Nosema* disease in North America is fumagillin, whose use is forbidden in Europe because it has no established Maximun Residue Level (MRL). With prolonged use of fumagillin, the issue of *Nosema* resistance to treatment will certainly arise [62–69]. Our study showed that the negative impacts of *Nosema* infection on the honey bee immune function and lifespan were exacerbated by antibiotic treatment because of the effects on the gut microbiota.

Collectively, the results of this study emphasize the importance of commensal gut bacteria in regulating honey bees’ susceptibility to *N*. *ceranae* infection, the risk of the disruption of gut bacteria by antibiotics in altering the proper function of host immune system, and the urgency of developing new disease therapeutic strategies that can help ameliorate the destruction of beneficial bacteria by antibiotics thus promoting honeybee health. A better understanding of how specific commensal microbes interact with the host immune system in the future may lead to potential therapeutic avenues for improving bee health.

## Supporting information

S1 Table Results of independent t- test of bacteria activity in honeybee of 3, 7 and 11 days post treatment. (PDF)

S2 Table Paired t- test result of bacteria phylotypes in honeybee of 3, 7 and 11 days post treatment. (PDF)

S3 Table One–Way ANOVA result of three antimicrobial peptides (AMPs) gene’s expression of *abaecin*, *defensin1*, and *hymenoptaecin* in honeybee of 3, 7 and 11 days post treatment respectively. (PDF)

S4 Table Independent t- test result of *N*. *ceranae* quantity in honeybee of 3, 7 and 11 days post treatment respectively. (PDF)
