# Supplementary material for: New evidence showing that the destruction of gut bacteria by antibiotic treatment could increase the honey bee’s vulnerability to Nosema infection
Source: PLoS One. 2017 Nov 10;12(11):e0187505. doi: 10.1371/journal.pone.0187505 (PMC5681286; doi:10.1371/journal.pone.0187505)
Supplement: S1 Table — (PDF) [file pone.0187505.s001.pdf]

Group Statistics

	group	N	Mean	Std. Deviation	Std. Error Mean
3 days	Treated	24	0	3.435222146	0.701211784
	No-treated	24	9.994870789	1.334432036	0.284501866
7 days	Treated	24	0	2.05411178	0.428311914
	No-treated	24	14.20128263	1.25422012	0.273693268
11 days	Treated	24	0	3.582786453	0.731333222
	No-treated	24	14.83541765	1.30385122	0.271871773

Independent Samples Test

		Levene's Test for Equality of Variances		t-test for Equality of Means						
		F	Sig.	t	df	Sig. (2-tailed)	Mean Difference	Std. Error Difference	95% Confidence Interval of the Difference	
									Lower	Upper
3 days	Equal variances assumed	13.42	0.001	-11.56	22	0	-9.039774545	0.781956898	-10.61570512	-7.463843967
	Equal variances not assumed			-11.946	11.432	0	-9.039774545	0.75672933	-10.58458819	-7.494960897
7 days	Equal variances assumed	2.517	0.12	-26.854	22	0	-13.94301477	0.519208816	-14.99082058	-12.89520896
	Equal variances not assumed			-27.431	18.629	0	-13.94301477	0.508290371	-14.97302959	-12.91299995
11 days	Equal variances assumed	11.8289919	0.001268015	-17.36452802	22	0	-13.77594207	0.793338123	-15.37380707	-12.17807706
	Equal variances not assumed			-17.65620434	11.526	0	-13.77594207	0.780232365	-15.37119112	-12.18069301
