# Supplementary material for: New evidence showing that the destruction of gut bacteria by antibiotic treatment could increase the honey bee’s vulnerability to Nosema infection
Source: PLoS One. 2017 Nov 10;12(11):e0187505. doi: 10.1371/journal.pone.0187505 (PMC5681286; doi:10.1371/journal.pone.0187505)
Supplement: S2 Table — (PDF) [file pone.0187505.s002.pdf]

Paired Samples Statistics

		Mean	N	Std. Deviation	Std. Error Mean
3 days	Group I	5.2853	8	3.16932	1.12052
	GroupII	5.4367	8	3.09846	1.09547
7 days	Group I	4.8526	8	4.04708	1.43086
	GroupII	4.7341	8	4.25804	1.50544
11 days	Group I	5.1237	8	4.52292	1.59909
	GroupII	5.5389	8	4.70875	1.66479

Paired Samples Correlations

		N	Correlation	Sig.
3 days	GroupI & Group II	8	.995	.000
7 days	GroupI & Group II	8	.985	.000
11 days	GroupI & Group II	8	.965	.000

Paired Samples Test

		Paired Differences					t	df	Sig. (2-tailed)
		Mean	Std. Deviation	Std. Error Mean	95% Confidence Lower	95% Confidence Upper			
3 days	GroupI - Group II	-.15142	.33307	.11776	-.42987	.12703	-1.286	7	.239
7 days	GroupI - Group II	.11846	.75887	.26830	-.51597	.75289	.442	7	.672
11 days	GroupI - Group II	-.41517	1.23481	.43657	-1.44750	.61716	-.951	7	.373

Group I: negative control

GroupII: Nosema innoculated
