# Supplementary material for: New evidence showing that the destruction of gut bacteria by antibiotic treatment could increase the honey bee’s vulnerability to Nosema infection
Source: PLoS One. 2017 Nov 10;12(11):e0187505. doi: 10.1371/journal.pone.0187505 (PMC5681286; doi:10.1371/journal.pone.0187505)
Supplement: S3 Table — (PDF) [file pone.0187505.s003.pdf]

Descriptives

		N	Mean	Std. Deviation	Std. Error	95% Confidence Interval for Mean		Minimum	Maximum
						Lower Bound	Upper Bound		
abaecin	Group I	12	5.7134	.95047	.31682	4.9828	6.4440	4.20	7.18
	Group II	12	3.4960	1.42136	.47379	2.4034	4.5885	.00	4.83
	Group III	12	1.2297	1.29352	.39001	.3607	2.0987	.02	3.58
	Group IV	12	1.0000	.45365	.14346	.6755	1.3245	.39	2.01
	Total	48	2.7285	2.18878	.35049	2.0190	3.4380	.00	7.18
defensin1	Group I	12	4.2110	.71527	.20648	3.7565	4.6654	2.67	5.15
	Group II	12	2.2997	.58071	.18364	1.8843	2.7151	1.07	2.95
	Group III	12	3.0034	1.20284	.34723	2.2392	3.7677	1.67	5.63
	Group IV	12	1.0000	.21886	.06599	.8530	1.1470	.68	1.40
	Total	48	2.6793	1.40559	.20953	2.2570	3.1016	.68	5.63
hymenoptaecin	Group I	12	6.1982	3.70894	1.07068	3.8417	8.5548	1.37	12.54
	Group II	12	3.0559	1.82254	.57634	1.7521	4.3597	.80	5.81
	Group III	12	3.6039	1.09433	.38690	2.6891	4.5188	1.63	5.32
	Group IV	12	1.0000	.63317	.19091	.5746	1.4254	.20	1.99
	Total	48	3.5310	2.96539	.46312	2.5950	4.4670	.20	12.54

Test of Homogeneity of Variances

	Levene Statistic	df1	df2	Sig.
abaecin	2.620	3	44	.066
defensin1	5.932	3	44	.002
hymenoptaecin	6.073	3	44	.002

ANOVA

		Sum of Squares	df	Mean Square	F	Sig.
abaecin	Between Groups	140.076	3	46.692	38.935	.000
	Within Groups	41.973	44	1.199		
	Total	182.049	47			
defensin1	Between Groups	61.874	3	20.625	33.748	.000
	Within Groups	25.057	44	.611		
	Total	86.930	47			
hymenoptaecin	Between Groups	158.136	3	52.712	10.074	.000
	Within Groups	193.606	44	5.233		
	Total	351.741	47			

Post Hoc Tests

Multiple Comparisons

	(I) Group	(J) Group		Mean Difference (I-J)	Std. Error	Sig.	95% Confidence Interval Lower Bound	95% Confidence Interval Upper Bound
abaecin	Tukey HSD	Group I	Group II	2.21742	.51623	.001	.8252	3.6096
			Group III	4.48373	.49221	.000	3.1563	5.8112
			Group IV	4.71339	.50316	.000	3.3564	6.0704
		Group II	Group I	-2.21742	.51623	.001	-3.6096	-.8252
			Group III	2.26631	.49221	.000	.9389	3.5937
			Group IV	2.49597	.50316	.000	1.1390	3.8529
		Group III	Group I	-4.48373	.49221	.000	-5.8112	-3.1563
			Group II	-2.26631	.49221	.000	-3.5937	-.9389
			Group IV	.22966	.47848	.963	-1.0608	1.5201
		Group IV	Group I	-4.71339	.50316	.000	-6.0704	-3.3564
			Group II	-2.49597	.50316	.000	-3.8529	-1.1390
			Group III	-.22966	.47848	.963	-1.5201	1.0608
	Dunnett T3	Group I	Group II	2.21742	.56996	.009	.4942	3.9406
			Group III	4.48373	.50248	.000	3.0105	5.9570
			Group IV	4.71339	.34779	.000	3.6243	5.8025
		Group II	Group I	-2.21742	.56996	.009	-3.9406	-.4942
			Group III	2.26631	.61366	.011	.4504	4.0822
			Group IV	2.49597	.49503	.003	.8941	4.0978
		Group III	Group I	-4.48373	.50248	.000	-5.9570	-3.0105
			Group II	-2.26631	.61366	.011	-4.0822	-.4504
			Group IV	.22966	.41556	.993	-1.0457	1.5050
		Group IV	Group I	-4.71339	.34779	.000	-5.8025	-3.6243
			Group II	-2.49597	.49503	.003	-4.0978	-.8941
			Group III	-.22966	.41556	.993	-1.5050	1.0457
defensin1	Tukey HSD	Group I	Group II	1.91123	.33473	.000	1.0150	2.8075
			Group III	1.20756	.31915	.003	.3530	2.0621
			Group IV	3.21096	.32632	.000	2.3372	4.0847
		Group II	Group I	-1.91123	.33473	.000	-2.8075	-1.0150
			Group III	-.70367	.33473	.169	-1.5999	.1926
			Group IV	1.29973	.34157	.003	.3851	2.2143
		Group III	Group I	-1.20756	.31915	.003	-2.0621	-.3530
			Group II	.70367	.33473	.169	-.1926	1.5999
			Group IV	2.00341	.32632	.000	1.1296	2.8772
		Group IV	Group I	-3.21096	.32632	.000	-4.0847	-2.3372
			Group II	-1.29973	.34157	.003	-2.2143	-.3851
			Group III	-2.00341	.32632	.000	-2.8772	-1.1296
	Dunnett T3	Group I	Group II	1.91123	.27633	.000	1.1106	2.7119
			Group III	1.20756	.40398	.044	.0236	2.3915
			Group IV	3.21096	.21677	.000	2.5503	3.8717
		Group II	Group I	-1.91123	.27633	.000	-2.7119	-1.1106
			Group III	-.70367	.39280	.407	-1.8662	.4589
			Group IV	1.29973	.19513	.000	.6897	1.9098
		Group III	Group I	-1.20756	.40398	.044	-2.3915	-.0236
			Group II	.70367	.39280	.407	-.4589	1.8662
			Group IV	2.00341	.35344	.001	.9065	3.1003
		Group IV	Group I	-3.21096	.21677	.000	-3.8717	-2.5503
			Group II	-1.29973	.19513	.000	-1.9098	-.6897
			Group III	-2.00341	.35344	.001	-3.1003	-.9065

hymenoptaecin	Tukey HSD	Group I	Group II	3.14236	.97944	.014	.5079	5.7768
			Group III	2.59431	1.04409	.079	-.2140	5.4027
			Group IV	5.19825	.95485	.000	2.6299	7.7666
		Group II	Group I	-3.14236	.97944	.014	-5.7768	-.5079
			Group III	-.54805	1.08505	.957	-3.4666	2.3705
			Group IV	2.05589	.99947	.186	-.6325	4.7442
		Group III	Group I	-2.59431	1.04409	.079	-5.4027	.2140
			Group II	.54805	1.08505	.957	-2.3705	3.4666
			Group IV	2.60394	1.06290	.085	-.2550	5.4629
		Group IV	Group I	-5.19825	.95485	.000	-7.7666	-2.6299
			Group II	-2.05589	.99947	.186	-4.7442	.6325
			Group III	-2.60394	1.06290	.085	-5.4629	.2550
	Dunnett T3	Group I	Group II	3.14236	1.21594	.104	-.4524	6.7371
			Group III	2.59431	1.13844	.195	-.8573	6.0459
			Group IV	5.19825	1.08757	.003	1.8183	8.5782
		Group II	Group I	-3.14236	1.21594	.104	-6.7371	.4524
			Group III	-.54805	.69416	.959	-2.6260	1.5299
			Group IV	2.05589	.60713	.033	.1474	3.9644
		Group III	Group I	-2.59431	1.13844	.195	-6.0459	.8573
			Group II	.54805	.69416	.959	-1.5299	2.6260
			Group IV	2.60394	.43144	.001	1.2340	3.9738
		Group IV	Group I	-5.19825	1.08757	.003	-8.5782	-1.8183
			Group II	-2.05589	.60713	.033	-3.9644	-.1474
			Group III	-2.60394	.43144	.001	-3.9738	-1.2340

Group I: Negative control
Group II: Nosema
Group III: Antibiotics
Group IV: Nosema + Antibiotics
