# Supplementary material for: New evidence showing that the destruction of gut bacteria by antibiotic treatment could increase the honey bee’s vulnerability to Nosema infection
Source: PLoS One. 2017 Nov 10;12(11):e0187505. doi: 10.1371/journal.pone.0187505 (PMC5681286; doi:10.1371/journal.pone.0187505)
Supplement: S4 Table — (PDF) [file pone.0187505.s004.pdf]

Group Statistics

		N	Mean	Std. Deviation	Std. Error Mean
3 days	Group IV	9	6.2327	3.91660	1.30553
	Group II	9	.0000	1.48576	.49525
7 days	Group IV	9	17.4536	1.18064	.39355
	Group II	9	8.1595	1.58551	.52850
11 days	Group IV	9	17.6869	1.24093	.50661
	Group II	9	7.1522	.87254	.29085

Independent Samples Test

		Levene's Test for Equality of Variances		t-test for Equality of Means						
									95% Confidence Interval of the Difference	
		F	Sig.	t	df	Sig. (2-tailed)	Mean Difference	Std. Error Difference	Lower	Upper
3 days	Equal variances assumed	8.800	.009	4.464	16	.000	6.23267	1.39631	3.27262	9.19273
	Equal variances not assumed			4.464	10.256	.001	6.23267	1.39631	3.13198	9.33337
7 days	Equal variances assumed	.602	.449	14.105	16	.000	9.29412	.65894	7.89724	10.69100
	Equal variances not assumed			14.105	14.786	.000	9.29412	.65894	7.88786	10.70039
11 days	Equal variances assumed	.165	.691	19.407	16	.000	10.53464	.54283	9.36193	11.70735
	Equal variances not assumed			18.034	8.277	.000	10.53464	.58416	9.19537	11.87391

Group IV: Nosema + Antibiotics

Group II: Nosema
